# Risks of miscarriage and inadvertent exposure to artemisinin derivatives in the first trimester of pregnancy: a prospective cohort study in western Kenya

**DOI:** 10.1186/s12936-015-0950-6

**Published:** 2015-11-18

**Authors:** Stephanie Dellicour, Meghna Desai, George Aol, Martina Oneko, Peter Ouma, Godfrey Bigogo, Deron C. Burton, Robert F. Breiman, Mary J. Hamel, Laurence Slutsker, Daniel Feikin, Simon Kariuki, Frank Odhiambo, Jayesh Pandit, Kayla F. Laserson, Greg Calip, Andy Stergachis, Feiko O. ter Kuile

**Affiliations:** Liverpool School of Tropical Medicine, Pembroke Place, Liverpool, L3 5QA UK; Centers for Disease Control and Prevention, Atlanta, GA USA; Kenya Medical Research Institute Centre for Global Health Research, Kisumu, Kenya; Global Health Institute, Emory University, Atlanta, GA USA; Bayer Healthcare, Nairobi, Kenya; Pharmacy Systems, Outcomes and Policy Department, University of Illinois at Chicago, Chicago, USA; Departments of Pharmacy and Global Health, Schools of Pharmacy and Public Health, University of Washington, Seattle, USA

**Keywords:** Anti-malarials, Pharmacovigilance, Drug safety in pregnancy, Teratogenicity, Miscarriage

## Abstract

**Background:**

The artemisinin anti-malarials are widely deployed as artemisinin-based combination therapy (ACT). However, they are not recommended for uncomplicated malaria during the first trimester because safety data from humans are scarce.

**Methods:**

This was a prospective cohort study of women of child-bearing age carried out in 2011–2013, evaluating the relationship between inadvertent ACT exposure during first trimester and miscarriage. Community-based surveillance was used to identify 1134 early pregnancies. Cox proportional hazard models with left truncation were used.

**Results:**

The risk of miscarriage among pregnancies exposed to ACT (confirmed + unconfirmed) in the first trimester, or during the embryo-sensitive period (≥6 to <13 weeks gestation) was higher than among pregnancies unexposed to anti-malarials in the first trimester: hazard ratio (HR) = 1.70, 95 % CI (1.08–2.68) and HR = 1.61 (0.96–2.70). For confirmed ACT-exposures (primary analysis) the corresponding values were: HR = 1.24 (0.56–2.74) and HR = 0.73 (0.19–2.82) relative to unexposed women, and HR = 0.99 (0.12–8.33) and HR = 0.32 (0.03–3.61) relative to quinine exposure, but the numbers of quinine exposures were very small.

**Conclusion:**

ACT exposure in early pregnancy was more common than quinine exposure. Confirmed inadvertent artemisinin exposure during the potential embryo-sensitive period was not associated with increased risk of miscarriage. Confirmatory studies are needed to rule out a smaller than three-fold increase in risk.

**Electronic supplementary material:**

The online version of this article (doi:10.1186/s12936-015-0950-6) contains supplementary material, which is available to authorized users.

## Background

Artemisinin-based combination therapy (ACT) anti-malarials have been adopted as first‐line treatment for falciparum malaria in almost all endemic countries, providing life‐saving benefits to children, adults and pregnant women globally [1]. However, their safety is uncertain when used in early pregnancy. Ascertainment of risk from exposure to anti-malarials in the first trimester is difficult in resource-poor settings and data available for assessing risk are limited [2, 3]. Artemisinins are embryo-toxic in several animal species, including non-human primate models [4, 5]. Teratogenic effects observed in mice and rabbits included death of the foetus, malformations of the heart, great vessels, and limb defects. Primate models exposed to prolonged courses of ACT had high rates of foetal loss [6]. Animal models suggested that artemisinin embryo-toxicity targets primitive erythroblasts, which are the primary form of red blood cells in circulation between weeks 4 and 10 post-conception in humans. Therefore the embryo-sensitive period to artemisinin, if any, is thought to occur at 6–12 (inclusive) weeks’ gestation from the first day of the last menstrual period (LMP) in humans [4, 5, 7, 8].

There are limited data available to assess whether ACT is embryo-toxic or teratogenic in humans; fewer than 700 exposures in the first trimester have been well documented [9–15]. After reviewing all existing evidence in 2003 and then in 2006, the World Health Organization (WHO) recommended that artemisinins could be used during the second or third trimesters of pregnancy and that, due to insufficient safety data, treatment in the first trimester was not recommended unless the life of the mother is at risk, or oral quinine is not available [5, 16]. The recommended treatment for first trimester malaria infections is seven days’ oral quinine alone or combined with clindamycin [17]. However, as women may not be aware of their pregnancy or do not declare an early pregnancy, and because clinic staff do not often assess for pregnancy in women of child-bearing age (WOCBA), the risk of exposure to drugs not recommended in pregnancy, including to potential teratogens, is possible during this period [18]. As ACT is increasingly available, a growing number of women will be inadvertently exposed to an artemisinin compound in early pregnancy, including during the period when foetal organs and tissues are formed.

Malaria can have severe consequences to the health of the pregnant woman and her unborn baby including maternal anaemia, foetal loss, preterm birth, low birth weight and perinatal mortality, and in some cases maternal death. The impact of malaria infection in early pregnancy has been identified as a major knowledge gap for estimating the burden of malaria in pregnancy. Recent studies have provided insight into the potential adverse consequences of malaria infections early in pregnancy, showing a major impact on birth weight and maternal anaemia [19, 20]. Findings from a retrospective analysis from 25 years of data from the Thai-Myanmar border, where artemisinin deployment has been necessary for many years because of multi-drug resistance, showed that malaria infection in the first trimester (both symptomatic and asymptomatic) was a significant risk factor for miscarriage. No association between first trimester artemisinin exposure and miscarriage was found. However more data from a wider range of malaria-endemic countries are required to provide an increased level of reassurance that first trimester artemisinin exposure does not significantly increase the risk of miscarriage or other adverse pregnancy outcomes. The findings from a prospective cohort study of WOCBA designed to examine whether ACT exposure in the first trimester was associated with miscarriage are reported here.

## Methods

### Overview of study design

This was a prospective cohort study conducted among WOCBA (15–49 years of age) residing in a highly malarious area in western Kenya with a population under continuous health and demographic surveillance system (HDSS) monitoring as part of the collaboration between the Kenya Medical Research Institute (KEMRI) and Centers for Disease Control and Prevention (CDC) [21]. Participants received treatment through the usual channels, including health facilities and drug outlets.

## Procedures

### Recruitment of women of child-bearing age and pregnancy detection

Between 15 February, 2011 and 15 February, 2013, 6010 WOCBA participating in an ongoing population-based, infectious disease, surveillance project (PBIDS) in rural Bondo District, western Kenya [22, 23] (Additional file 1) were invited to participate in the ‘Evaluation of Medications used in Early Pregnancy’ (EMEP) prospective cohort study. EMEP staff visited all homes in the PBIDS and enrolled consenting WOCBA who met eligibility criteria for EMEP. WOCBA were eligible for EMEP if they were between 15 and 49 years of age and active participants of PBIDS. Exclusion criteria included: inability to give informed consent or provide an accurate medical history. WOCBA who consented to participate were asked if they could be pregnant and offered a pregnancy test at the time of enrolment and again approximately every 3 months thereafter. Any participant with a detected pregnancy was referred to the antenatal clinic at Lwak Hospital where trained EMEP nurses confirmed the pregnancy (either by ultrasound if the women presented before 24 weeks, or by palpation and by auscultation of the foetal heart later in pregnancy) and offered free antenatal care (ANC). Additionally, all pregnant patients presenting at the ANC clinic of Lwak Hospital were enrolled if all criteria were met. EMEP nurses were not involved in treatment of study participants.

### Gestational age assessment

Gestational age was determined using the most accurate measurement available for each participant in the following order: ultrasound scan taken before 24 weeks’ gestation performed by trained study nurses (Sonosite 180 plus portable ultrasound system), Ballard estimates measured within 96 h of birth, LMP or reported gestation at time of pregnancy loss, and, lastly gestational age derived from fundal height assessment (Additional file 1).

### Pregnancy outcome

Pregnancy outcomes were assessed using a combination of health facility- and home-based follow-up visits. The latter is particularly relevant for miscarriages, because the vast majority of these events occur in the community, not in health facilities. Village-based staff received monthly lists of participants with estimated delivery dates and after visiting the participants’ homes they informed study nurses of pregnancy outcomes. Follow-ups by study staff were then arranged to administer structured questionnaires about the delivery, outcome, any illnesses and medication used during pregnancy. Pregnancy outcomes captured included: pregnancy loss (miscarriages, induced abortions and stillbirths), live births and major congenital malformations detectable at birth by surface examination. This analysis focuses on miscarriage defined as spontaneous pregnancy loss at or before 28 completed weeks’ gestation (2–28 weeks inclusive), which is considered the gestational age of viability in resource-constrained settings.

### Anti-malarial drug exposure ascertainment

Drug exposure data were captured using three approaches (Table 1): (a) interviews with pregnant women visiting the antenatal clinic in Lwak Hospital and at the time of pregnancy outcome follow-up (henceforth referred to as EMEP data); (b) record linkage to data on drugs prescribed to WOCBA at the outpatient department in Lwak Hospital (henceforth referred to as Lwak-OPD data); and, (c) weekly to twice monthly home visits by fieldworkers as part of PBIDS.

**Table 1:** Description of drug information sources used to determine anti-malarial and malaria exposure status

Approach	Format	Drug information available
EMEP self-report	Retrospective self-report of illness and medication used since the beginning of the pregnancy collected at every ANC visit and at pregnancy outcome follow-up visit. A general open question about any drug use as well as a directed question for specific anti-malarials were included as using medication/indication-specific questions have been shown to improve accuracy. Photographs of all anti-malarial drugs found in the study area were used to facilitate recognition of drug names. A calendar marking public holidays and school closures was also used to enhance recall of dates	Drug name Drug start date Duration Number of tablets per day Indication and indication diagnosis Drug source
Lwak-OPD records	Prospective documentation by health facility clinic staff of diagnosis and treatment prescribed at outpatient department (OPD) whenever a PBIDS participant sought care at Lwak Hospital for an infectious syndrome	Date of visit Diagnosis Prescribed treatment
PBIDS weekly and twice-monthly home visits	Self-report of symptoms, health-seeking behaviour and medication. This information was collected continuously on a weekly (from 5 January, 2010 to 26 May, 2011) and then twice-monthly basis (27 May, 2011 onwards). The same visual aids as described above were used for recall of drug intake	Date of visit Symptoms in previous week/2 weeks Treatment taken for the symptoms including drug name If and where care was sought

*ANC* antenatal care, *EMEP* evaluation of medications used in early pregnancy study, *Lwak OPD* Lwak hospital out-patient department, *PBIDS* population-based infectious disease surveillance

### Other covariates

Obstetric history and ANC laboratory information collected routinely at antenatal booking (haemoglobin level, HIV and syphilis testing, and malaria microscopy) were extracted from the ANC records at Lwak hospital or antenatal cards by study nurses. Demographic characteristics and medical history, including illnesses (e.g., malaria) and drugs used during the current pregnancy were collected at each EMEP study visit at ANC and during pregnancy outcome follow-up visits. Household level wealth quintiles were obtained from the HDSS [24].

### Data analysis

#### Exposure definition

A trend of increase in risk of miscarriage with ACT exposure during this artemisinin-specific, embryo-sensitive period would corroborate the biological mechanism observed in animal models and suggest a causal association with ACT exposures. The analysis focused on two exposure definitions: anti-malarial drug reported/prescribed (1) ‘anytime’ in the first trimester, i.e., gestational week 2 and 0 days (day 14 since LMP) to week 13 and 6 days (day 97 since LMP) post-LMP, and (2) between weeks 6 day 0 (day 42 since LMP) to week 12 day 6 post-LMP (day 90 since LMP) (potential artemisinin embryo-sensitive period as suggested by animal reprotoxicology [8]). Unexposed was defined as no evidence of anti-malarial or malaria exposure in any of the three data sources. Confirmed exposures were defined as exposures identified by at least two of the three data sources. Confirmed + unconfirmed exposures were defined as anti-malarial identified by at least one of the three data sources.

#### Cox regression model

Analyses were performed using Stata v12.1 (StataCorp LP, College Station, TX, USA). Cox proportional hazard regression models with left truncation were fitted to estimate the effect on miscarriage of ACT exposure during the first trimester and during the artemisinin embryo-sensitive period. Exposure was treated as a time-dependent variable (Additional file 1). Known risk factors for miscarriage were considered and to determine which variables remained in the final model, assessment of confounding was based on the impact a variable had on the hazard ratio, followed by the consideration of its precision. If the HR changed by ≥10 % the variable was retained in the model [25, 26].

The primary analysis compared the hazard of miscarriage among pregnancies with confirmed ACT exposures, either anytime during the first trimester or six to 12 weeks post-LMP, with the hazard among women not exposed to any anti-malarials anytime during the first trimester or among women exposed to quinine anytime in the first trimester or 6–12 weeks post-LMP.

Secondary analyses consisted of similar models but using (a) less restrictive exposure definitions, including both confirmed and unconfirmed exposures, and, (b) more restrictive exposure definitions where only ACT exposures within estimated gestational age confidence margins were included (Additional file 2).

### Ethical review and consent

The EMEP study was approved by the ethics committees and institutional review boards of CDC (No. 5889), KEMRI (No. 1752) and the Liverpool School of Tropical Medicine (No. 09.70). Written informed consent or assent was obtained from each participant including consent for record linkage with PBIDS and HDSS databases.

## Results

### Participant characteristics

**Fig. 1:**
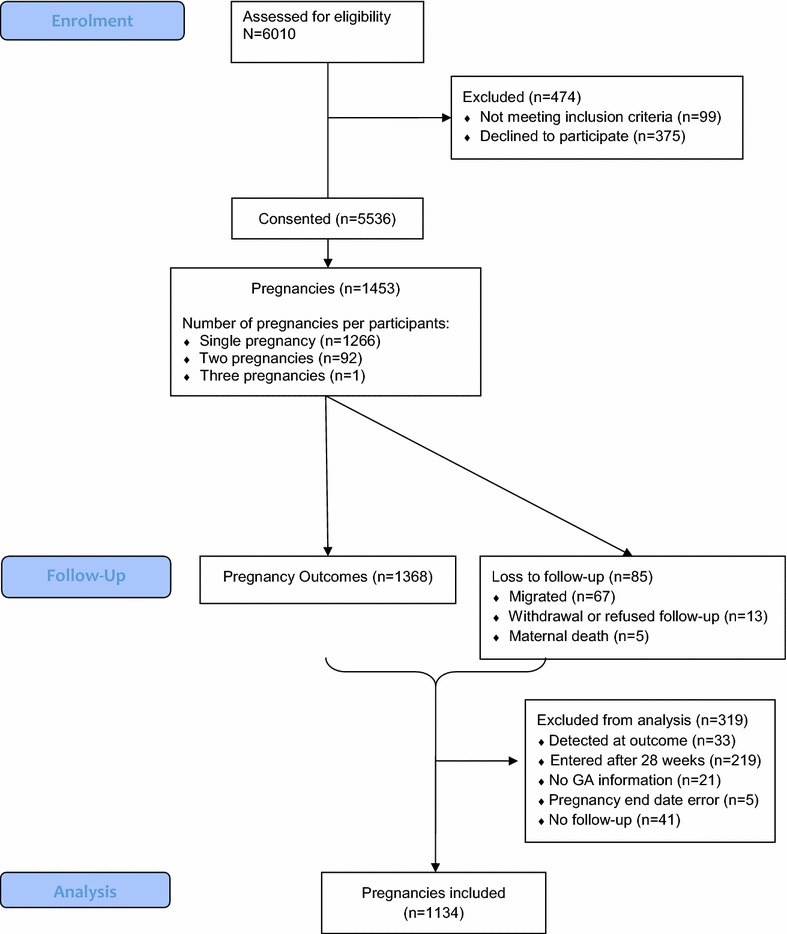
Study participant flow diagram from screening to inclusion in data analysis

Out of 5911 eligible WOCBA, 5536 (94 %) consented to participate and among them, 1453 pregnancies were detected, and 1134 (78 %) were included in the data analysis (Fig. 1). The mean and median gestational age at time of pregnancy detection was 13.3 (standard deviation 6.9) and 12.1 (range 0–27.9) weeks (Table 2). Overall, 62 % of deliveries took place at a health facility, and 25 % of the miscarriages. Overall, 67 % of pregnancy outcomes were captured within a week of the event; however, for miscarriages this was only 20 %.

**Table 2:** Characteristics of 1134 pregnancies by ACT exposure status [n (%) otherwise stated]

	Overall (N = 1134)	No ACT exposure in the first trimester (N = 835)	Unconfirmed ACT exposure in the first trimester (N = 222)	Confirmed ACT exposure in the first trimester (N = 77)	P values*
Age in years [mean (SD; range)]	26.1 (6.8; 15–47)	26.1 (6.7; 15–45)	26.7 (7.2; 15–47)	25.2 (6.5; 16–41)	0.225
Gravidity	Missing n = 16	Missing n = 14	Missing n = 1	Missing n = 1	0.065
Primigravidae	219 (19.6)	151 (18.4)	47 (21.3)	21 (27.6)	
1–3 pregnancies	525 (47.0)	405 (49.3)	90 (40.7)	30 (39.5)	
4+ pregnancies	374 (33.5)	265 (32.3)	84 (38.0)	25 (32.9)	
Previous pregnancy loss	160 (14.3), Missing n = 17	118 (14.4), Missing n = 15	30 (13.6), Missing n = 1	12 (15.8), Missing n = 1	0.888
Gestational age at detection in weeks [mean (SD; range)]^a^	13.3 (6.9; 0–27.9)	13.3 (7.0; 0–27.9)	13.0 (6.7; 0.3–27)	13.6 (7.1; 2.4–27.4)	0.770
Occupation	Missing n = 31	Missing n = 28	Missing n = 1	Missing n = 2	0.191
Not working	379 (34.4)	281 (34.8)	68 (30.8)	30 (40.0)	
Farming	369 (33.5)	268 (33.2)	80 (36.2)	21 (28.0)	
Small business/Skilled Labour	335 (30.4)	246 (30.5)	65 (29.4)	24 (32.0)	
Other	20 (1.8)	12 (1.5)	8 (3.6)	0	
Antenatal care summary					
Number of ANC visit	Missing n = 39	Missing n = 31	Missing n = 5	Missing n = 3	0.125
None	89 (8.1)	64 (8.0)	21 (9.7)	4 (5.4)	
1	90 (8.2)	61 (7.6)	24 (11.1)	5 (6.8)	
2	155 (14.2)	121 (15.1)	25 (11.5)	9 (12.2)	
3	244 (22.3)	193 (24.0)	38 (17.5)	13 (17.6)	
4+	517 (47.2)	365 (45.4)	109 (50.2)	43 (58.1)	
Gestational age at first ANC visit in weeks [mean (SD)]*	20.8 (7.8) range: 1.7–41.0	21.24 (7.8) range: 2.7–41.0	19.7 (7.6) range: 1.7–41.0	19.4 (7.7) range: 3.4-37.0	0.020
HIV positive^b^	Missing n = 101	Missing n = 79	Missing n = 18	Missing n = 4	0.354
Negative	771 (74.4)	562 (74.3)	149 (73.0)	60 (82.2)	
Positive	262 (25.4)	194 (25.7)	55 (27.0)	13 (17.8)	

*ACT* artemisinin combination therapy, *SD* standard deviation

* P values refer to Pearson Chi square test for categorical variables and ANOVA test for continuous variables

^a^Gestational age lowest estimate include 0 which reflects inaccuracy in the gestational age measurements

^b^HIV status information was not available for 12 % (129) of pregnancies that did not attend antenatal care or have the antenatal card for review. HIV status information was complemented by HDSS and data which offered home-based HIV testing and counselling to PBIDS participants. Test results were linked to the study participants using unique ID and missing data were updated if the test was performed before the pregnancy detection for HIV positive test results and for HIV negative results if the test was performed maximum 3 months before or after pregnancy detection. An additional 30 HIV status were ascertained while 8 % (99) still had no HIV status data

### Prevalence of first trimester ACT and quinine exposure

**Fig. 2:**
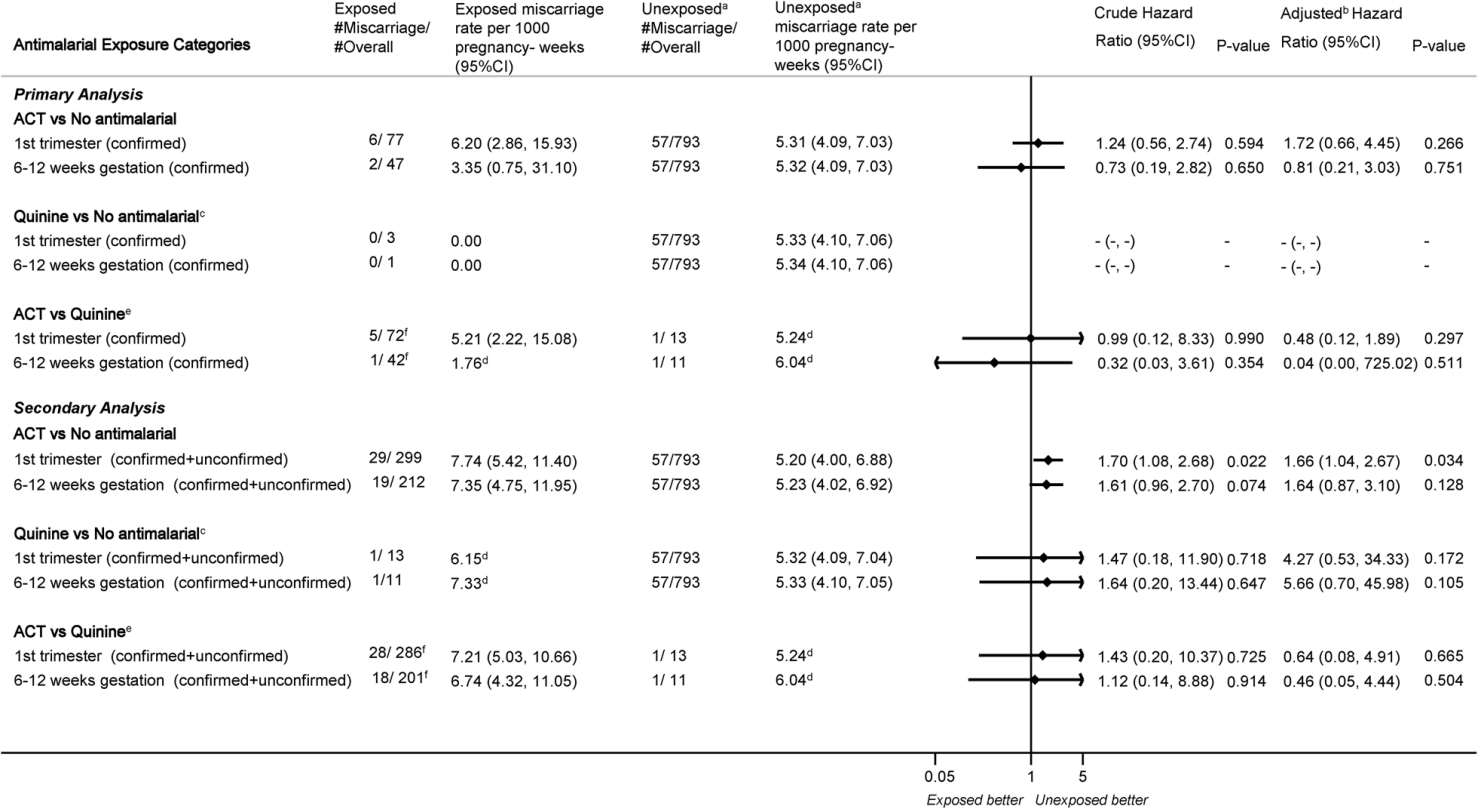
Miscarriage rate, unadjusted and adjusted hazard rates for the association between different anti-malarial exposure categories and miscarriage

Overall, 299 (26.4 %) of the 1134 pregnancies had evidence of possible ACT exposure anytime in the first trimester (confirmed + unconfirmed). For 77 (25.8 % of exposures and 6.8 % of all pregnancies) this could be confirmed by at least two of the three sources; 56 of these confirmed exposures (18.7, 5.3 % of pregnancies) were within the estimated gestational age confidence margins. For 212 out of 299 first trimester exposures (70.9 %, 18.7 % of pregnancies), the exposure occurred between 6 and 12 weeks’ gestation; 47 of them were confirmed exposures (Fig. 2). Only 13 pregnancies were exposed to quinine-alone anytime in the first trimester, and 11 during the 6–12 weeks’ gestational period.

### Association between first trimester ACT-exposure and miscarriage

#### Confirmed exposure (primary analysis)

Compared to pregnancies without anti-malarial exposure/malaria in the first trimester (793), the hazard for miscarriage was non-significantly higher among women with confirmed ACT exposures anytime in the first trimester (77) [hazard ratio (HR) = 1.24, 95 % CI (0.56–2.73)], and this was HR = 1.72 (0.66–4.45) in multivariate analysis (Fig. 2). The corresponding values for ACT exposure during the embryo-sensitive period (47) were HR = 0.73 (0.19–2.82) and HR = 0.81 (0.21–3.03) (Fig. 2).

The values when compared against quinine (13) were: HR = 0.99 (0.12–8.33) and HR = 0. 32 (0.03–3.61) (crude analysis) for exposure anytime and six to 12 weeks post-LMP (Fig. 2).

More restrictive definitions to define exposure within the redefined margins for gestational age resulted in similar or lower effect estimates, but numbers of exposures and events were limited (Additional file 2). The method used for missing value did not alter the conclusions (Additional file 3).

### Confirmed + unconfirmed exposure (secondary analysis)

When using a less restrictive definition of exposure by including unconfirmed exposures as well, the risk of miscarriages was significantly higher among the ACT-exposed pregnancies relative to unexposed pregnancies: adjusted HR = 1.66 (1.04–2.67). This was HR = 1. 61 (0.96–2.70) for the embryo-sensitive period. The HRs when compared to quinine were HR = 0.64 (0.08–4.91) and HR = 0.46 (0.05–4.44), respectively (Fig. 2).

## Discussion

Pregnancies exposed to ACT in the first trimester were at increased risk of miscarriage compared to pregnancies not exposed to anti-malarials in the same gestation period. This was only statistically significant at the 5 % level in the group with the less restrictive definition for exposure (confirmed and unconfirmed) which had higher number of events (29) and exposures (299) [adjusted HR = 1.66 95 % CI (1.04–2.67)]. A similar effect measure [HR = 1.72 95 % CI (0.66–4.45)] was obtained when the analysis was restricted to those exposures that could be confirmed by the OPD database or the ongoing household surveillance, which was the primary analysis. However the available exposures (77) and events (six) were reduced markedly with this more restrictive analysis and the difference was not statistically significant. When the analysis was further restricted to exposures in the potential embryo-sensitive period in humans for the artemisinins, the effect estimates were again similar [HR = 1. 61 95 % CI (0.96, 2.70)] for confirmed + unconfirmed exposures, but much lower for confirmed exposures [HR = 0.73 95 % CI (0.19, 2.82)]. However this latter analysis, which was also part of the primary analysis, included only two events and 47 pregnancies exposed to ACT. There was no evidence for an increase in the risk of miscarriage among women treated with ACT *versus* women treated with oral quinine, but again the number exposed to quinine alone was limited to 13 with only one miscarriage.

It was expected that the risk of miscarriage would be higher among women who received anti-malarials than among women without anti-malarial exposure early in pregnancy. This is related to the potential for confounding by indication, i.e., women treated with ACT or quinine sought treatment because of their malaria or other febrile illness, whereas women who did not require anti-malarials did not. The comparison with untreated women is therefore difficult to interpret as it does not allow for the differentiation between the effects of malaria and the drug treating it. Malaria itself, even if it remains asymptomatic, is a known cause of miscarriage. A recent meta-analysis of five trials with malaria chemoprophylaxis or intermittent preventive therapy in 2876 *paucigravidae* in sub-Saharan Africa showed that women in the control arms were at a 1.54 95 % CI (0.98–2.44) higher risk of miscarrying than women protected by chemoprevention [27]. Prospective studies in low malaria-transmission areas in Thailand also found that asymptomatic malaria in the first trimester increased the odds of miscarriage nearly three-fold and symptomatic infections four-fold [13]. The 1.4- to 1.7-fold increased risk for miscarriage among women exposed to ACT or quinine relative to pregnancies not requiring treatment observed in this study is thus within the expected range of malaria-associated risk of miscarriage.

This study is underpowered to confidently detect or exclude effects smaller than a three-fold increased risk of miscarriage associated with ACT. Nevertheless no indication for such a potential association was found. First, there was no indication that the effect size associated with ACT exposure relative to unexposed women was greater among women treated during the embryo-sensitive period than at anytime during the first trimester. If ACT was causing miscarriage through this mechanism, the effect size would be expected to be highest for exposures restricted to that embryo-sensitive period. No such trend was observed. Secondly, the rates of miscarriage in the quinine-only and ACT-exposed pregnancies were similar. Although the comparison with quinine needs to be interpreted with caution due to the small numbers of quinine-only exposed women, these results are consistent with observations from the Thai-Burmese border by McGready et al. They also found no difference in the proportions of pregnancies ending in miscarriages between women treated with chloroquine (26 %), quinine (27 %) or artesunate (31 %) [13]. A recent prospective study from Tanzania reported higher risk of pregnancy loss (miscarriage and stillbirth combined) in women exposed to quinine compared to those exposed to ACT [14]. A prospective study in Zambia found higher occurrence of miscarriage in first trimester ACT-exposed pregnancies (5 %) compared to none in those exposed to sulfadoxine-pyrimethamine or quinine but the number exposed to quinine (six) were too small to allow for a meaningful comparison [12].

The small number of quinine exposures in the first trimester in this study was surprising as this is the recommended first-line malaria treatment in the first trimester. However these observations are consistent with a recent study on malaria in pregnancy-prescribing practice carried out in the same area of western Kenya (Riley et al., unpublished) and a study from Uganda [28]. These studies draw attention to the need to assess reasons for poor adherence to quinine and malaria treatment guidelines. Poor tolerability and poor compliance to its seven-day regimen is a known problem for treatment of malaria with oral quinine [29, 30].

This study had several limitations that should be considered. First, the small number of quinine exposures limited the ability to compare ACT-exposed pregnancies to the purported ‘control’ drug (as quinine is not known to cause miscarriages) [3]. Second, it was not possible to control for confounding by indication (i.e., the disease itself) because laboratory confirmation of malaria was not available for most women. Controlling for malaria and its severity is important, as malaria itself has been suggested to reduce the potential risk of embryo-toxicity from artemisinin as was found in rat models [31]. Third, since induced abortions are illegal in Kenya, this could have resulted in induced abortions being reported as miscarriages. However since neither ACT nor quinine exposures are perceived as indications for induced abortion in this population, it is thus unlikely that such misclassification would differ according to exposure status. Fourth, it was not possible to account for exposure misclassification due to lack of adherence to prescribed medication (drug intake was not observed) or from counterfeit anti-malarials [32], which could bias the estimate towards the null. Fifth, the ability to confirm exposure was limited because there was limited overlap in the exposures ascertained in the three data sources. The group at highest risk for bias are the unconfirmed exposure cases as 32 first-trimester, ACT-exposures were only reported after pregnancy outcome. Recall bias following adverse pregnancy outcome has been well documented, hence the focus in this study was to confirm ACT exposures using prospective drug ascertainment approaches through record linkage to minimize such bias [33–35]. Another potential source of exposure misclassification is gestational age measurement errors. The study could not assess any dose–response effect of exposure.

## Conclusion

The results presented here are consistent with two previous observational studies showing an increased risk of miscarriage among women treated for malaria with ACT in the first trimester *versus* unexposed women, and a similar [13] or lower risk compared to oral quinine [14]. These results also suggest that ACT use in the first trimester is much more common than quinine. The risk associated with malaria in early pregnancy, the comparable observed risk between ACT and quinine exposures, and the limited compliance to treatment with quinine suggests a trial comparing ACT *versus* quinine for the treatment of uncomplicated malaria in the first trimester may be merited. Before such a trial is considered, further safety data on the association between ACT and congenital malformations, that is forthcoming from studies conducted by the Malaria in Pregnancy Consortium and WHO, should be reviewed and all available evidence pooled to evaluate the evidence of the risk and benefits of artemisinin use in early pregnancy.

## Additional files

10.1186/s12936-015-0950-6 Detailed description of study site and methodology.
10.1186/s12936-015-0950-6 Description of sensitivity analysis looking at potential gestational age measurement error.
10.1186/s12936-015-0950-6 Description of sensitivity analysis using multiple imputation for missing data.

## Abbreviations

ACT: artemisinin-based combination therapy
ANC: antenatal care
CDC: US Centers for Disease Control and Prevention
EMEP: evaluation of medications used in early pregnancy study
HDSS: health and demographic surveillance system
HR: hazard ratio
KEMRI: Kenya Medical Research Institute
LMP: last menstrual period
OPD: outpatient department
PBIDS: population-based infectious disease surveillance project
WHO: World Health Organization
WOCBA: women of childbearing age
