# Supplementary material for: Risks of miscarriage and inadvertent exposure to artemisinin derivatives in the first trimester of pregnancy: a prospective cohort study in western Kenya
Source: Malar J. 2015 Nov 18;14:461. doi: 10.1186/s12936-015-0950-6 (PMC4652370; doi:10.1186/s12936-015-0950-6)
Supplement: Supplementary file 1 — 10.1186/s12936-015-0950-6 Detailed description of study site and methodology. [file 12936_2015_950_MOESM1_ESM.docx]

# Supplemental File S1. Detailed description of study site and methodology

Contents

[The study site and population 2](#_Toc419728370)

[Community mobilization and formative research 3](#_Toc419728371)

[Staffing and Field infrastructure 4](#_Toc419728373)

[Data collection 5](#_Toc419728374)

[*Enrolment* 5](#_Toc419728375)

[*Pregnancy detection strategy* 5](#_Toc419728376)

[*Pregnancy follow up* 6](#_Toc419728377)

[*Gestational age assessment* 6](#_Toc419728378)

[*Source of information on drug exposure* 6](#_Toc419728379)

[*Malaria infection* 7](#_Toc419728380)

[*Pregnancy outcome assessment* 7](#_Toc419728381)

[Data Analysis 8](#_Toc419728382)

[Quality Control 9](#_Toc419728383)

[*Data Management* 9](#_Toc419728384)

[Enrolment and Pregnancy Detection 9](#_Toc419728385)

[*Newborn exam* 9](#_Toc419728386)

[*Ultrasound* 10](#_Toc419728387)

[References 10](#_Toc419728388)

[Acronyms 11](#_Toc419728389)

## The study site and population

The study area was located in Rarieda District in Siaya County, lying northeast of Lake Victoria in Nyanza Province, western Kenya. The cohort was carried out in 33 adjacent villages in the Asembo area (Figure S1.1). The study area comprises one mission hospital, 2 government-run health centres and 4 dispensaries. The provincial teaching hospital is located in Kisumu town approximately 50 km away and the closest district hospital is Bondo District Hospital 13km away. This area is under continuous health and demographic surveillance (HDSS) by Kenya Medical Research Institute (KEMRI) and the Centers for Disease Control and Prevention (CDC) Research and Public Health Collaboration since 2001. [[1](#_ENREF_1), [2](#_ENREF_2)] Through quarterly household visits, information on births, deaths, and migrations are collected. Cause of death is established through verbal autopsy. In addition to the HDSS, the 33 villages are under enhanced morbidity surveillance since 2005 through the KEMRI/CDC International Emerging Infection program (IEIP) to investigate major infectious disease syndromes. This program has been described in details elsewhere. [[3](#_ENREF_3), [4](#_ENREF_4)] As part of this population-based infectious disease surveillance project (PBIDS), trained fieldworkers visit households regularly (on a weekly basis from January 5, 2010 to May 26, 2011 and then bi-weekly basis from May 27, 2011 onwards) collecting information on all illnesses since the previous visit, the source of care and any medication taken, including specific antimalarial medication. In addition all visits for infectious symptoms Lwak Mission Hospital, the referral health facility for PBIDS, are recorded (including diagnosis made and prescriptions given) using the HDSS ID for record linkage. All care for acute illness is free at this health facility for PBIDS study participants, thereby enhancing health utilization. The morbidity data is linked to socio-economic and demographic data collected by the HDSS. The HDSS and IEIP platform provided a unique opportunity to monitor drug use in pregnancy and develop/validate methodology for pharmacovigilance throughout the pregnancy period.

Rainfall is bimodal with long rains between March-June and short rains in October-December. Average temperature ranges between 18C and 36C at a mean altitude of approximately 1070 metres above sea level. Over 95 percent of the inhabitants are from the Luo ethnic group. The majority of the population is Christian and there is a small Muslim community. Polygamy is common with about 21% of women in Nyanza province in a polygamous relationship.[[5](#_ENREF_5)] The main income generating activities are fishing, subsistence farming and cattle keeping and small scale businesses. Houses are typically made out of mud, brick or cement and roof are either made of thatched grass or iron sheets. The majority have completed primary school education but only 23% finished secondary school. The total population in the Asembo area of the HDSS was 64,491 with 22% women of childbearing age (15-49 years) in 2010. The total fertility rate for 2009 was estimated at 4.3 in this area. Migrations are significant and the rates female out- and in-migration for females in Asembo were 127 and 111 per 1000 person year respectively in 2009.[[6](#_ENREF_6)]

Nyanza province has a high burden of disease and worst health indicators compared to the overall Kenyan national statistics.[[5](#_ENREF_5)] Malaria transmission is perennial and holo-endemic with peaks following the two rainy seasons. Annual cross-sectional survey in this area showed parasitaemia of 42% in under-5 years old, 60% in 5-14 years old and 20% in over 14 year old (unpublished KEMRI/CDC data for 2010). Whereas the national HIV prevalence is 6.3% (4% for men and 8% for women), the prevalence for Nyanza province is close to double around 14% (11% for men and 16% for women).[[7](#_ENREF_7)] In Asembo, infant mortality was 94 per 1000 live-births and under five mortality ratio was 181 per 1000 live-births (unpublished HDSS fact sheet for 2009).

## Community mobilization and formative research

The success and acceptability of any study is highly dependent on efforts made to involve the community and provide enough information in an accessible manner. Community mobilisation activities started in September 2010. A series of meetings were held with District Medical Officer for Health (DMOH), the village chiefs, district officers and counsellors, the community advisory board (CAB) set up by KEMRI/CDC and community members to introduce and get feedback on the proposed study plans. “Barazas” (community meetings) where held in the pilot villages in November and in each IEIP study villages in February and March 2011. Study brochures were also distributed at the community meetings and at the central health facility. Formative research involving 10 focus group discussions was carried out in September 2010 with the aim to explore the socio-cultural context around pregnancy and to investigate acceptability of proposed study procedures[[8](#_ENREF_8), [9](#_ENREF_9)]. A pilot study was subsequently carried out in 3 villages in November-December to assess implementability and identify bottlenecks before wider implementation. Overall positive feedback was received through community meetings and acceptability during the pilot was high. Some concerns were raised regarding the need for the study to provide free ANC, delivery and referral services. Lessons learned in terms of field logistics were incorporated for the study implementation which started on the 15^th^ of February 2011. Free ANC services were offered at the study health facility but covering delivery and full referral services was beyond the scope of this study and this was clarified at Barazas before implementation. Participants presenting to Lwak Mission Hospital for their ANC received a free delivery kit which entitled them to reduced costs of delivery at the same health facility (from KES 900 to KES 500 for a standard delivery).

##
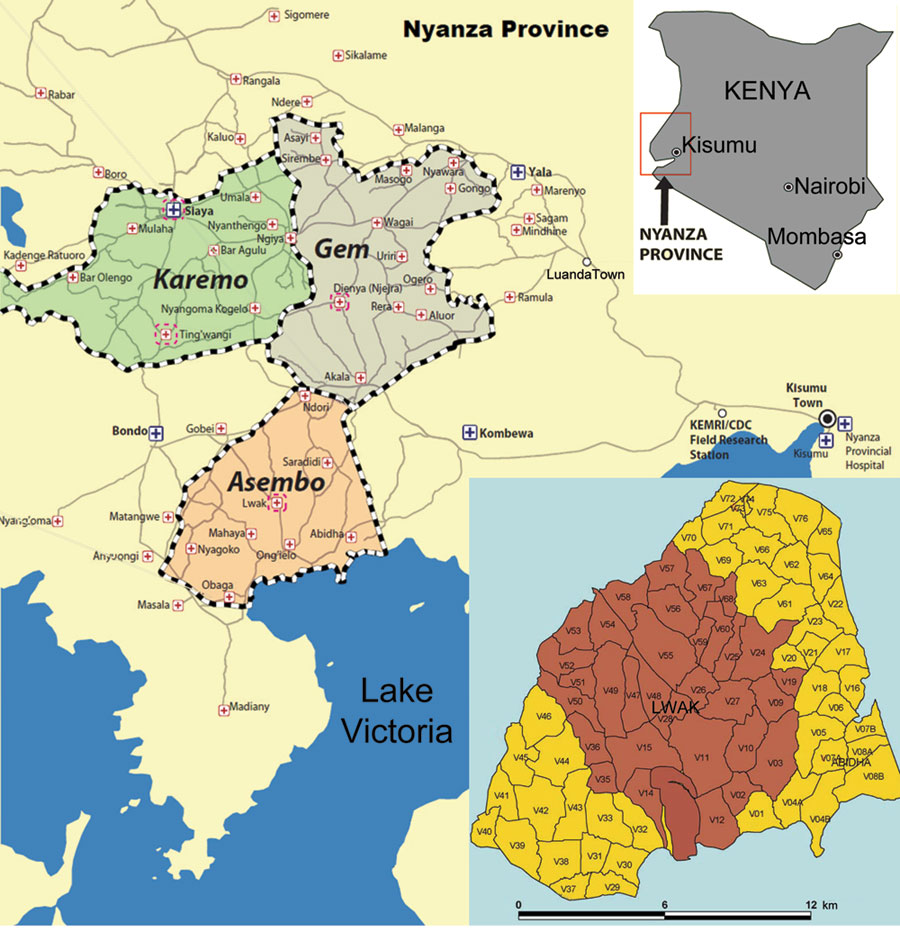


Figure S1.1. Map of study area

## Staffing and Field infrastructure

The core study team comprise of a study coordinator, a field supervisor, two field assistants and three study nurses. All went through one week training covering Good Clinical Practice (GCP), research ethics and consenting procedures, fieldwork etiquette and interview techniques, as well as protocol and study tools (including training on the use of the netbook for data collection). In addition to the overall protocol training, study nurses were also trained on newborn examination for recognition of congenital malformations detectable by surface examination and Ballard score for gestational assessment (2 days) using training documents developed by the Malaria in Pregnancy Consortium (MIPc), use of ultrasound for gestational assessment (5 days) and a 2-week motorbike training for home follow ups.

A team of 40 village based staff called “village reporters” or VRs were involved for the community mobilisation, enrolment and pregnancy detection activities. VRs usually act as community liaison officers for various KEMRI/CDC projects, some of them have been involved in research since the pivotal bednet trial which was carried out in Asembo and Gem in 1998-2001.[[10](#_ENREF_10)] All the 40 VRs involved in the study were females. They received a 3-day training giving an overview of research ethics, the study (background, objective, procedure, expected output and role and responsibilities), consenting process and pregnancy test procedure, counselling and referral. A one day refresher was provided before initiation of each village and each VR was accompanied by the supervisor or field assistants during the first few visits.

## Data collection

### *Enrolment*

Following community mobilisation, door-to-door enrolment was carried out to provide information about the study to eligible women of childbearing age (15-49 years). All female age 15-49 years residents of households within the defined catchment area and participating PBIDS [[3](#_ENREF_3)] were eligible for enrolment in the study. Women were excluded if they refused to participate; were unable to provide informed consent due to mental, physical or social inability or if they refused to be followed up at the end of the pregnancy. A list of all PBIDS female participants within the specified age group was provided to the VRs. Women above 18 years of age and mature minors (defined as an under 18 year old who is either married, head of a household or already a parent) could provide consent for themselves. Parental consent and assent were sought for minors (<18 years of age not qualifying as a mature minor). Information on screening and enrolment was collected on simple scannable forms in addition to the signed informed consent forms. Enrolment was active throughout the study period (from mid-February 2011 to mid-February 2013) whereby newly eligible women (turned 15 years of age or in-migrant joining PBIDS) could be enrolled at any time. Additionally, all pregnant patients presenting at the antenatal clinic (ANC) at Lwak Hospital were enrolled if all criteria were met.

### *Pregnancy detection strategy*

At the time of enrolment, all consented participants were asked about their pregnancy status and offered to take a pregnancy test if they were not visibly pregnant. Following the initial enrolment period, VRs visited all study participants every three months at their home to collect information on their pregnancy status and offer pregnancy testing. The three-monthly home visits started in October 2011. In between home visits the VRs also kept a stock of test strips, gloves and urine cups and participants were made aware that they could access pregnancy tests through the VRs or the study health facility any time they wished. Although some pregnancies (30) were detected at delivery, about half (45%) were detected in their first trimester and 69% were detected using a pregnancy test before being visible. The average gestational age at the time of pregnancy detection varied according to the approach (Figure S1.2): pregnancies detected at the time of enrolment in the study had an average gestational age of 17 weeks (range 0-42 weeks); pregnancies detected by pregnancy tests by VRs were detected around 16 weeks; whereas pregnancies detected at the ANC were on average 23 weeks (range 2-41 weeks).

Figure S1.2 Graph of the number of pregnancies detected by the different strategy and the line represents the average gestational age (in weeks) at detection over time.

### *Pregnancy follow up*

Pregnant participants were referred for free ANC at Lwak Mission Hospital where trained study nurses were running the antenatal clinic. Participants were managed according to the Ministry of Health guidelines for ANC including a full ANC profile (covering blood group, syphilis, urinalysis, haemoglobin and HIV) and in addition received ultrasound scan if they presented before 6 months of gestation. Following the ANC consultation the participant was asked a series of additional question for the study covering demographic characteristics, medical history and description of any illnesses and/or medication taken since the beginning of the index pregnancy. At each ANC revisits (scheduled according to national recommendation of minimum of four ANC visit per pregnancy), the participants were asked about any illnesses and treatment sought since their last ANC visits.

### *Gestational age assessment*

Gestational age was assessed using multiple methods and was determined using the most accurate measurement available for each participant. Ultrasound assessment of gestational age up to 24 weeks provides the most accurate prediction of the expected date of delivery, this was offered to all participants presenting at the study facility before 24weeks gestation. Ballard score was performed on all live newborns seen by a study nurse within seven days of birth but only estimates based on assessment within 96hours after birth were used to assess gestational age. In case of a pregnancy loss, the participant was asked if she knew how many months pregnant she was at the time of the loss, of whom 26% did and reported a gestational age. The first day of the last menstruation period (LMP) was asked to all participants at the time of pregnancy detection by trained VRs and was asked again during follow up visit by a study nurse either at ANC or at the time of outcome follow up (if the participant had not been seen at ANC at the study health facility). Eleven percent reported not to know the date of their LMP. Fundal height was also measured at each ANC visit at the study health facility and recorded by a trained study nurse. Only measurements done between 20 and 34 weeks gestation in singleton pregnancies were used and gestation estimated using the McDonald rule formula: # of cm x 8/7 = wks gestation. There were 15 participants (including a lost to follow up) for whom no gestational age data was available as the participants didn’t know their LMP, had no ultrasound scan, fundal height or Ballard Score that could be used.

### *Source of information on drug exposure*

Drug exposure data was captured using three approaches: a) interview of pregnant participants seen at the study facility ANC and at the time of pregnancy outcome follow up (from here on referred to as EMEP data); b) record linkage of data on all drugs prescribed to WOCBAs at outpatient department in Lwak hospital (from here on referred to as Lwak OPD data) and lastly c) via an active approach involving regular home visits by field workers as part of the IEIP morbidity study (from here on referred to as PBIDS data).

1. *EMEP self-reported drug exposure:* Pregnant participants attending ANC in Lwak were asked about drug exposure since the beginning of their pregnancy and at subsequent ANC visit about any drugs taken since their last visit. At the time of the pregnancy outcome follow up visit, participants were interviewed by a study nurse regarding drugs used in pregnancy and other medically relevant information. The limitation of drug histories is accuracy of recalled information. Visual aids depicting photographs of all antimalarial drugs found in the study area were used to facilitate recognition of drug names. Calendar marking public holidays and school closures was also used to enhance recall of dates.
2. *Lwak OPD Health Facility records:* The existing IEIP system in Lwak Mission Hospital is set up to document diagnosis and related treatments in all age-groups at OPD. All participants were already enrolled in the IEIP program and had access to free care at Lwak Mission Hospital for infectious syndrome.
3. *PBIDS (bi-)weekly home visits:* This active approach involved home visits by trained fieldworkers on a weekly (from January 5, 2010 to May 26, 2011) and then bi-weekly basis (May 27, 2011 onwards), before a woman might becomes aware of her pregnancy. The interview component on drug exposure was also enhanced by introducing visual aids for recall (same as the one mentioned above under EMEP self-reported drug exposure). This approach should limit drug exposure recall bias as the information is collected continuously (concurrently to when exposure occurs or close to this time) before a woman might become aware of her pregnancy. However a limitation of this approach is that interview data is incomplete for weeks when no one was found at home (54% of times) and on some occasion the interview was completed by a proxy (family member available on the day of the interview) instead of the participant herself.

None of these methods represent a “gold standard” to ascertain drug exposures. The Lwak OPD data source was limited to participants receiving treatment from this facility (only 40% EMEP ACT exposures in the 1^st^ trimester were reported to be from Lwak health facility and 48% of cases reporting taking an antimalarial at a (bi-)weekly home visit also sought care from Lwak health facility). Thus LWAK OPD data source was incomplete in terms of ACT received from other health facilities, drug outlets or relatives/friends and maybe misleading in a case if a woman was prescribed an antimalarial but did not take it. The limitation of the PBIDS home visits in part reflects the incompleteness of the data as 32% of participants did not have all home visits for the period of their 1^st^ trimester of pregnancy (due to instances when no one was at home). Further, 26% of first trimester visits were completed by proxy (i.e. a relative responding on behalf of the participant) which might not be as accurate and complete compared to data generated from interviews completed by participants. The EMEP data source is limited by the participant recall, however pictorial aids and calendars were used to facilitate better recall.

### *Malaria infection*

Information on episodes of malaria was captured through interviews by study nurses or through OPD records for those who sought care from Lwak Hospital. Reliable information on malaria diagnosis was only available from Lwak OPD which has the laboratory capacity to perform malaria slide microscopy.[[3](#_ENREF_3)] EMEP nurses asked about recent care-seeking and diagnosis for each reported illness including malaria episodes but reliable information on laboratory confirmed malaria was generally not available.

### *Pregnancy outcome assessment*

Pregnancy outcome captured included miscarriage, induced abortion (where possible), stillbirth, and live-birth. Miscarriage were defined as a confirmed pregnancy (positive pregnancy test and/or confirmed at by nurse at ANC) that fails to progress, resulting in death and expulsion of the embryo or unviable fetus (< 28 weeks for developing countries). Induced abortions were defined as artificial interruption of pregnancy. Stillbirth was defined as a pregnancy lasting until at least 28 weeks of gestation that results in the birth of a stillborn infant.

A combination of facility-based and home-based follow-up systems were used to ascertain pregnancy outcomes because the majority of deliveries occurred in the home (only 44% delivered in a health facility according to KDHS 2008/9). This is particularly important for miscarriages, the vast majority of which occur outside health facilities. Therefore, a home-based follow up system was developed, using VRs that alerted their supervisor by phone regarding any pregnancy outcome. VRs were incentivised to report any pregnancy outcome as soon as possible after delivery (they received Ksh120 for pregnancy outcomes reported within 4 days and Ksh 90 otherwise based on the standard rate for birth notification at the time) through the KEMRI/CDC HDSS). VRs received monthly lists of participants with estimated delivery dates in their respective catchment area. Simple scannable forms including the expected date of delivery (based on ultrasound EDD if available otherwise on LMP) were distributed to the field team as a prompt that a delivery was expected. Participants were encouraged and counselled on the benefits of delivering at a health facility although the study did not cover delivery costs. At their last ANC visit, participants received a simple delivery kit (including sterile gloves, gauze, cord clamp, soap, razor blade, maternity pads and clear plastic sheet) to bring to the health facility which provided a discount on their delivery costs. Once notified about a pregnancy outcome the VR supervisor coordinated the follow up to be conducted by a study nurse either at home or at the health facility. A detailed questionnaire about the delivery, outcome and newborn examination findings was administered in addition to the questions regarding illnesses/medication used during pregnancy. This took approximately 60min. If the mother consented, the baby was photographed. Any case of suspected malformation or abnormality was reviewed by the study paediatrician either by going through the newborn exam data and the photos or by full physical review if the latter information was inconclusive. Cases needing further investigation or intervention were referred to the appropriate facilities/ specialist.

## Data Analysis

Analyses were performed using Stata v12.1 (StataCorp LP, College Station, Texas). Cox proportional hazard regression models with left truncation were fitted to estimate the effect on miscarriage of ACT exposure during the 1^st^ trimester and during the artemisinin embryo-sensitive period. Since women were exposed to ACTs at different gestational ages in the 1st trimester, ACT exposure was treated as a time-dependent variable. For example, if a woman took an ACT during gestation week 9, she was considered unexposed during the first weeks and then exposed from week 9 onwards.[[11-13](#_ENREF_11)] The reference group consisted of pregnancy-weeks without exposure to antimalarials or malaria during the entire 1^st^ trimester according to any of the 3 exposure data sources. The proportional hazard (PH) assumption was assessed for each covariate using a graphical approach (the log-log Kaplan-Meier survival plots assessing if the curves for different strata are parallel) and based on Schoenfeld residuals for the global test, and Schoenfeld scaled residuals for separate tests with each covariate. This showed that the proportionality assumption was not violated for any of the main effect and covariates in the final models. We assumed left truncation was non-informative since none of the risk factors for miscarriage were associated with time of pregnancy detection (data not shown). The cluster function for robust standard errors in Stata was used to account for the fact that some participants had multiple pregnancies during the study period. Pregnancies that ended in induced abortions (9), withdrawal (4) or loss to follow up before 28 weeks (20) were censored at the time of that event, or at the time of their last follow-up visit in case of loss to follow-up.

We considered the following potential confounding factors based on known risk factors for miscarriage as well as common socioeconomic variables: maternal age at the time of pregnancy detection[[14](#_ENREF_14), [15](#_ENREF_15)]; gravidity[[16](#_ENREF_16)]; previous pregnancy loss[[17](#_ENREF_17)]; socioeconomic characteristics (education, marital status and occupation [[18](#_ENREF_18), [19](#_ENREF_19)]). If values for potential co-variates were missing for <10% of pregnancies they were imputed using multiple imputations to create fifty data sets. Sensitivity analysis was conducted to explore the effect of the method used to include missing data by comparing results obtained through multiple imputation with results obtained when creating a category ‘unknown’ for those with missing value. Although syphilis[[20](#_ENREF_20)], and anaemia (defined as haemoglobin <11g/dl) could be potential confounders they were not considered in the full models because information was lacking for 29.9% to 27.2% of the pregnancies. Missing data were particularly frequent for miscarriage cases due to the fact that most of these pregnancies (74%) did not persist long enough to attend ANC (mean gestational age for 1^st^ ANC visit 20.8 weeks and mean gestational age for pregnancy loss 14.4 weeks). No women reported they smoked, while alcohol consumption (7), diabetes (2) and epilepsy (5) were infrequent and subsequently these factors were not included in further analysis. Malaria infection was not considered in the multivariate model since information on diagnosis was not consistently available plus malaria was strongly correlated with the antimalarial drug exposure of interest. To determine which variables remained in the final model, assessment of confounding was based on the impact a variable had on the hazard ratio, followed by the consideration of its precision. If the HR changed by >=10% the variable was retained in the model. Variables identified as non-confounders were dropped from the model provided their deletion led to a gain in precision for the effect estimate by examining confidence intervals.[[21](#_ENREF_21), [22](#_ENREF_22)]

## Quality Control

### *Data Management*

Enrolment and Pregnancy Status (paper forms)

All scannable forms were verified in the field by the study assistants when returned by the VRs before being sent for scanning at the KEMRI/CDC offices in Kisian. Once the forms arrived in Kisian they were scanned by the data management team who verified the entries on the computer screen before uploading the data to the database. The forms were filled in secure office space for easy retrieval in case of any query or data correction needed.

Data captured at ANC visits and Pregnancy Outcome follow up (electronic data capture)

All data collected by the study nurses for pregnancy follow up at the ANC or at outcome follow up were captured electronically on a Netbook computer with an in-house program for data collection developed using visual studio 2008 (VB.net programming language) and stored on MSSQL SERVER 2005/2008 on the Netbooks and Ms Access for the central database held in the KEMRI/CDC office. The data entry system contained in-built skip pattern depending on the entry, data checks for inconsistencies (i.e. data out of expected range) and certain fields which could not be left blank. Hard copies of the questionnaire were available in case the netbook had a problem. The data were backed up daily on SD-cards in the field and were brought to the central database in Kisian where they were downloaded once a week.

### Enrolment and Pregnancy Detection

A sample of eligible participants from each village was selected for re-visits by field assistants every 3 months following VRs visit. The data collected by the VR were compared to the data collected by the field assistant to verify that 1) the participant had been visited, 2) had been explained the study adequately and 3) the data collected on the form were correct.

### *Newborn exam*

On a bi-weekly basis two study nurses would independently carry out a newborn and Ballard score examinations for the same baby and compare their findings on the forms provided. Any discrepancy was discussed at the time and the baby re-examined to identify the correct value. In case were there was ambiguity about a correct value further explanation was sought from the study paediatrician and refresher training on Ballard assessment was organised.

### *Ultrasound*

Quality control for ultrasound scans was implemented during the pre-implementation phase following the ultrasound training. All ultrasound images were downloaded from the machine to a laptop and sent to ultrasound expert at the collaborating institution at the University of Washington (after removing all identifiers from the images). The expert reviewers provided feedback on a formatted excel spreadsheet and highlighted scans that were incorrect as well as areas needing re-training or special attention. If required, additional training materials were sent for review with the study nurses.

**Ethical review and consent**

The EMEP study protocol was reviewed and approved by the institutional review boards of CDC (No. 5889), KEMRI (No. 1752) and the Liverpool School of Tropical Medicine (No. 09.70). Informed written consent was obtained from each participant. For illiterate participants, an independent witness had to be there during the consenting process and sign the consent form while the participant marked the form with a thumbprint. Minors (girls under 18 years of age) needed parental consent before being asked for assent. Mature minor (defined as an under 18 year old who is either married, head of a household or already a parent) provided consent for themselves.

## References

1. Adazu K, Lindblade KA, Rosen DH, et al. Health and demographic surveillance in rural western Kenya: a platform for evaluating interventions to reduce morbidity and mortality from infectious diseases. Am J Trop Med Hyg **2005**; 73(6): 1151-8.

2. Odhiambo FO, Laserson KF, Sewe M, et al. Profile: the KEMRI/CDC Health and Demographic Surveillance System--Western Kenya. Int J Epidemiol **2012**; 41(4): 977-87.

3. Feikin DR, Audi A, Olack B, et al. Evaluation of the optimal recall period for disease symptoms in home-based morbidity surveillance in rural and urban Kenya. Int J Epidemiol **2010**; 39(2): 450-8.

4. Bigogo G, Audi A, Aura B, Aol G, Breiman RF, Feikin DR. Health-seeking patterns among participants of population-based morbidity surveillance in rural western Kenya: implications for calculating disease rates. Int J Infect Dis **2010**; 14(11): e967-73.

5. Kenya National Bureau of Statistics (KNBS) and ICF Macro. Kenya Demographic and Health Survey 2008-09. Calverton, Maryland, USA. <http://www.measuredhs.com/pubs/pdf/FR229/FR229.pdf>, **2011**.

6. KEMRI/CDC. Health and Demographic Surveillance System Report for 2009. Kisian: KEMRI/CDC, **2010**.

7. Kenya National Bureau of Statistics (KNBS), Macro I. Kenya Demographic and Health Survey 2008-09. Calverton, Maryland, **2010.**

8. Dellicour S, Desai M, Aol G, et al. Risks of miscarriage and inadvertent exposure to artemisinin derivatives in the first trimester of pregnancy: a prospective study in western Kenya. In preparation.

9. Mason L., Dellicour S., Ter Kuile F., et al. Barriers and facilitators to antenatal and delivery care in western Kenya: a qualitative study. BMC Pregnancy and Childbirth **2015**; 15(26).

10. Phillips-Howard PA, Nahlen BL, Kolczak MS, et al. Efficacy of permethrin-treated bed nets in the prevention of mortality in young children in an area of high perennial malaria transmission in western Kenya. Am J Trop Med Hyg **2003**; 68(4 Suppl): 23-9.

11. Howards PP, Hertz-Picciotto I, Poole C. Conditions for bias from differential left truncation. Am J Epidemiol **2007**; 165(4): 444-52.

12. Howards PP, Hertz-Picciotto I, Weinberg CR, Poole C. Misclassification of gestational age in the study of spontaneous abortion. Am J Epidemiol **2006**; 164(11): 1126-36.

13. Xu R, Luo Y, Chambers C. Assessing the effect of vaccine on spontaneous abortion using time-dependent covariates Cox models. Pharmacoepidemiol Drug Saf **2012**; 21(8): 844-50.

14. Nybo Andersen AM, Wohlfahrt J, Christens P, Olsen J, Melbye M. Maternal age and fetal loss: population based register linkage study. BMJ **2000**; 320(7251): 1708-12.

15. Smith KE, Buyalos RP. The profound impact of patient age on pregnancy outcome after early detection of fetal cardiac activity. Fertil Steril **1996**; 65(1): 35-40.

16. Kline J. I. An epidemiological review of the role of gravidity in spontaneous abortion. Early Hum Dev **1978**; 1(4): 337-44.

17. Regan L, Braude PR, Trembath PL. Influence of past reproductive performance on risk of spontaneous abortion. BMJ **1989**; 299(6698): 541-5.

18. Banerjee B, Dey TK, Chatterjee P. Work related physical exertion and spontaneous abortion. Indian J Public Health **2005**; 49(4): 248-9.

19. Kline JK. Maternal occupation: effects on spontaneous abortions and malformations. Occup Med **1986**; 1(3): 381-403.

20. Holder WR, Knox JM. Syphilis in pregnancy. Med Clin North Am **1972**; 56(5): 1151-60.

21. Kleinbaum DG, Klein M. Chapter 6: Modeling Strategy Guidelines. Logistic Regression: A Self-Learning Text. 3rd edition ed: Springer, **2010**.

22. Sonis J. A closer look at confounding. Fam Med **1998**; 30(8): 584-8.

## Acronyms

| **Acronym** | **Definition** |
| --- | --- |
| ACT | Artemisinin Combination Therapy |
| ANC | Antenatal Care |
| CAB | community advisory board |
| CDC | Centers for Disease Control and Prevention, Atlanta, USA |
| DMOH | District Medical Officer for Health |
| EMEP | Evaluation of Medicines used in Early Pregnancy study |
| GA | gestational age in weeks since last menstrual period |
| GCP | Good Clinical Practice |
| HDSS | health and demographic surveillance |
| IEIP | International Emerging Infection program |
| IPTp | Intermittent Presumptive Treatment in pregnancy |
| KEMRI | Kenya Medical Research Institute |
| LMP | Date of Last Menstrual Period |
| MiPc | Malaria in Pregnancy Consortium |
| MOH | Ministry of Health |
| OPD | Outpatient Department |
| PBIDS | population-based infectious disease surveillance project |
| TFR | total fertility rate |
| VR | Village Reporters |
| WOCBA | Women of childbearing age (15-49 years) |
