# Supplementary material for: Risks of miscarriage and inadvertent exposure to artemisinin derivatives in the first trimester of pregnancy: a prospective cohort study in western Kenya
Source: Malar J. 2015 Nov 18;14:461. doi: 10.1186/s12936-015-0950-6 (PMC4652370; doi:10.1186/s12936-015-0950-6)
Supplement: Supplementary file 2 — 10.1186/s12936-015-0950-6 Description of sensitivity analysis looking at potential gestational age measurement error. [file 12936_2015_950_MOESM2_ESM.docx]

# Supplemental File S3. Description of sensitivity analysis looking at potential gestational age measurement error.

We carried out a sensitivity analysis to look into the effect of potential exposure misclassification by assessing the impact of the measurement error in gestational age assessment. This analysis restricted ACT exposures to those within estimated gestational age confidence margins (excluding women whose exposure fell within the potential error margins for gestational age measurement). That is, to address the uncertainty related to the assessment of gestational age, we excluded pregnancies with exposures that could have occurred before conception or in the 2^nd^ trimester due to possible error in gestational age assessment. For example: an ACT exposure at 13 weeks estimated using the Ballard Score method has an estimated error margin of 2 weeks therefore the true gestational age probably lies between 11 and 15 weeks. Since the upper margin for this exposure would fall outside the 1^st^ trimester, such cases were excluded from the exposure group. Table S3a depicts the accuracy of each gestational age assessment method used and the corresponding expected error margin. The methods are listed in order of decreasing accuracy. In addition if the LMP agreed within 10 days of ultrasound, fundal height or Ballard estimates the error margin was reduced to +/- 10 days. Although the numbers of exposed and events were much lower and limited interpretation of this sensitivity analysis, the effect estimates were lower than effect estimates including all exposure anytime in the 1^st^ trimester or in the artemisinin embryo-sensitive period (Table S3b).

| **Table S3a. Accuracy correction for the gestational age assessment methods listed in order of decreasing accuracy.** | |
| --- | --- |
| **Method of Gestational Age Assessment** | **Accuracy Correction** |
| 1. Ultrasound before 14 gestational weeks | ± 5 days^a^ |
| 1. Ultrasound between 14 and 24 gestational weeks | ± 2 weeks^a^ |
| 1. Ballard score within 7 days of birth | ±2 week^b^ |
| 1. LMP/Self report GA at end of pregnancy | ± 4 weeks |
| 1. Fundal Height | ± 6 weeks- with multiple measurements ^c^ |

^a^Seffah JD, Adanu RM (2009) Obstetric ultrasonography in low-income countries. Clin Obstet Gynecol 52: 250-255.

^b^Sasidharan K, Dutta S, Narang A (2009) Validity of New Ballard Score until 7th day of postnatal life in moderately preterm neonates. Arch Dis Child Fetal Neonatal Ed 94: F39-44.

^c^White LJ, Lee SJ, Stepniewska K, Simpson JA, Dwell SL, et al. (2012) Estimation of gestational age from fundal height: a solution for resource-poor settings. J R Soc Interface 9: 503-510.

| **Table S3b. Miscarriage rate, unadjusted and adjusted hazard rates for the association between miscarriage and ACT exposure categories restricted to gestational age confidence margin.** | | | | | | | | |
| --- | --- | --- | --- | --- | --- | --- | --- | --- |
| Antimalarial Exposure Categories | Exposed #Miscarriage/ #Overall | Exposed Miscarriage rate per 1000 pregnancy-weeks (95%CI) | Unexposed #Miscarriage/ #Overall | Unexposed Miscarriage rate per 1000 pregnancy-weeks (95%CI) | Unadjusted Hazard Ratio (95%CI) | P-value | Adjusted Hazard Ratio (95%CI) | P-value |
| *ACT vs Unexposed to antimalarial* | | | | | | | | |
| 1st trimester (confirmed) | 3/ 56 | 4.09  (1.31, 19) | 57/793 | 5.32  (4.09, 7.04) | 0.85  (0.28, 2.56) | 0.774 | 1.05  (0.31, 3.56) | 0.940 |
| 6-12 weeks gestation (confirmed) | 0/ 22 | 0.00 | 57/793 | 5.33  (4.10, 7.05) | - (-, -) | - | - (-, -) | - |
| *ACT vs Quinine* | | | | | | | | |
| 1st trimester (confirmed) | 2/ 51 | 2.82  (0.63, 26.22) | 1/ 13 | 5.24 | 0.54  (0.06, 5.11) | 0.588 | - (-, -) | - |
| 6-12 weeks gestation (confirmed) | 0/ 21 | 0.00 | 1/ 11 | 6.04 | - (-, -) | - | - (-, -) | - |
