# Supplementary material for: Risks of miscarriage and inadvertent exposure to artemisinin derivatives in the first trimester of pregnancy: a prospective cohort study in western Kenya
Source: Malar J. 2015 Nov 18;14:461. doi: 10.1186/s12936-015-0950-6 (PMC4652370; doi:10.1186/s12936-015-0950-6)
Supplement: Supplementary file 3 — 10.1186/s12936-015-0950-6 Description of sensitivity analysis using multiple imputation for missing data. [file 12936_2015_950_MOESM3_ESM.docx]

# Supplemental File S2. Description of sensitivity analysis using multiple imputation for missing data.

As a sensitivity analysis we looked at the effect of using multiple imputation for the covariate in the model compared to using complete cases analysis or categorizing missing values as an “unknown” category. For the imputation of HIV status and occupation, we used information from all 1134 pregnancies and included gestational age at pregnancy detection, maternal age, gravidity, use of traditional remedies, education, marital status, household socio-economic group, previous pregnancy loss as well as ACT 1st trimester exposure status and miscarriage in the imputation model. We used imputation by chained equations and set the number of iterations to 50. Eighty eight out of the 101 missing HIV status were imputed and 15 out of 31 missing for occupation. As seen in the table below the effect estimates for ACT exposures were consistent for all adjusted models except for the complete case analysis. Since HIV status was disproportionally missing for miscarriage cases, which can be explained by the fact that most miscarriage occur before the mean gestational age for 1^st^ ANC visit in the study area when HIV status is usually determined. This model is most susceptible to selection bias by selecting out almost half of the events.

**Table S2: Comparison of the effect estimates for the risk of miscarriage in relation to Confirmed ACT 1^st^ trimester exposure in main and sensitivity analyses.**

|  | #Miscarriage/ # Overall Exposed | #Miscarriage/ # Overall  Unexposed | **HR** | **95%Confidence Interval** | **P-Value** |
| --- | --- | --- | --- | --- | --- |
| *Confirmed ACT 1st trimester vs No antimalarial exposures* | | | | |  |
| **Crude** | 6/77 | 57/793 | 1.24 | 0.56- 2.74 | 0.594 |
| Adjusted age occupation (complete case analysis)* | 6/75 | 54/766 | 1.56 | 0.70- 3.44 | 0.275 |
| Adjusted age occupation & HIV(complete case analysis) | 5/72 | 22/700 | 4.09 | 1.44- 11.57 | 0.008 |
| Adjusted age occupation (missing as unknown category) | 6/77 | 57/793 | 1.51 | 0.68- 3.32 | 0.309 |
| Adjusted age occupation & HIV (missing as unknown category) | 6/77 | 57/793 | 1.72 | 0.66- 4.45 | 0.266 |
| Adjusted age occupation (missing imputed)** | 6/76 | 57/779 | 1.50 | 0.68- 3.30 | 0.313 |
| Adjusted age occupation & HIV (missing imputed)** | 6/76 | 57/779 | 1.65 | 0.70- 3.89 | 0.255 |
| *Confirmed ACT 1st trimester vs Quinine* | | | | |  |
| **Crude** | 5/72 | 1/13 | 0.99 | 0.12- 8.33 | 0.990 |
| Adjusted age occupation (complete case analysis) | 5/68 | 1/13 | 1.46 | 0.37- 5.83 | 0.591 |
| Adjusted age occupation & HIV(complete case analysis) | 4/65 | 1/13 | 0.56 | 0.14- 2.22 | 0.412 |
| Adjusted age occupation (missing as unknown category) | 5/72 | 1/13 | 1.46 | 0.37- 5.83 | 0.591 |
| Adjusted age occupation & HIV (missing as unknown category) | 5/72 | 1/13 | 0.48 | 0.12- 1.89 | 0.297 |
| Adjusted age occupation (missing imputed)** | 5/69 | 1/13 | 1.44 | 0.36- 5.75 | 0.603 |
| Adjusted age occupation & HIV (missing imputed)** | 5/69 | 1/13 | 2.44 | 0.40- 14.99 | 0.336 |

* Note that in the analysis of complete cases only 27 out of 63 miscarriage events (less than half) are kept in the model

**For one ACT confirmed case and 14 unexposed cases missing values for occupation and HIV were not imputable as these were loss to follow-up with too many other missing data.
